# Computer‐Assisted Implant Surgery: Implications for Teaching, Learning, and Educational Strategies

**DOI:** 10.1002/cre2.70197

**Published:** 2025-07-31

**Authors:** Lin Jing Uei, Xin Hui Yeo, Yiu Yan Leung, George Pelekos, Bilal Al Nawas, Nikos Mattheos

**Affiliations:** ^1^ Department of Stomatology Ditmanson Medical Foundation Chia‐Yi Christian Hospital Chia‐Yi Taiwan; ^2^ Faculty of Dentistry Chulalongkorn University Bangkok Thailand; ^3^ The University of Hong Kong Hong Kong SAR China; ^4^ University Medical Center Mainz Mainz Germany; ^5^ Faculty of Dentistry and Digital Implant Surgery Research Unit Chulalongkorn University Bangkok Thailand; ^6^ Dental Department Karolinska Institute Stockholm Sweden; ^7^ Faculty of Dentistry The University of Hong Kong Hong Kong SAR China

**Keywords:** CAIS, dental education, dental implants, guided surgery, teaching and learning

## Abstract

**Objective:**

This white paper aimed to discuss educational strategies for teaching and learning of computer‐assisted implant surgery (CAIS) within the wider dental curriculum, including the pedagogy, technologies, and challenges.

**Material and Methods:**

A review of the available literature up to February 2025 was conducted, compiling existing evidence related to the application of CAIS in dental education, both through simulation and in clinical care. The findings were further integrated with expert opinions and current best practices in dental education in a panel discussion.

**Results:**

Guided CAIS demonstrated potential to enhance implant placement accuracy and increase confidence among novice surgeons. Dynamic CAIS (d‐CAIS) could support broader cognitive and surgical skill development, whereas static CAIS (s‐CAIS) showed limited utility as a comprehensive educational instrument. The sequence of learning between guided and non‐guided techniques appears to be less critical than ensuring proficiency in both. Competence development with d‐CAIS is incremental, unlike s‐CAIS, where progress might be faster but involves mainly procedural skills. Key challenges remain for the implementation of CAIS in educational structures such as limited faculty expertise, high costs of digital equipment, limited interoperability of digital systems, and ethical or legal concerns regarding autonomy of technologies.

**Conclusion:**

CAIS has shown to be a valuable tool in teaching implant dentistry; its proper integration into dental education enhances the learners' experience and offers significant benefits for developing overall surgical competences. Current educational research is limited and primarily focused on the accuracy of implant placement, while practice of CAIS entails a very wide set of parameters encompassing the clinician, the workflow, the institution or clinic, and the patient. Future studies targeting educational interventions with a wider array of outcome measures would be required to help design effective educational strategies with CAIS.

## 1 Introduction

Implant dentistry is being transformed with the introduction of digital technologies, but also transitioning towards new paradigms, such as design‐driven treatment plan (Pedrinaci et al. 2024; Mattheos et al. 2021), immediacy (Amorfini et al. 2017; Morton et al. 2023), and minimal invasiveness (Joda et al. 2018). Such paradigms are introducing new workflows, in which digital technologies play an increasingly critical part in the diagnosis, treatment plan, and execution of surgical and restorative procedures. Computer‐assisted implant surgery (CAIS) is one of the critical components of the digital workflows, which allows accurate implant placement in a position directed by the pre‐therapeutic design, thus empowering the personalized, design‐driven paradigm of implant dentistry (Pedrinaci et al. 2024; Mattheos et al. 2021).

The rapid transformation of implant care with the implementation of new digital devices and procedures poses significant educational challenges. Dentists caring for patients with implants should be familiar with modern digital workflows for the benefit of their patients due to the overall improved efficacy and quality of care. Currently practicing dentists will need support with clinically relevant, quality education and mentoring to upgrade their skills and implement the essential workflows in their everyday clinical practice. Design and provision of quality assured, continuous professional development (CPD) is critical to that end, as evidence suggests the majority of dentists acquire implant‐related competences outside university education (Dragan et al. 2019). At the same time, undergraduate and post‐graduate university education needs to be updated, so that those currently under training will develop essential skills on the digital workflows, devices, and procedures which they will be most likely to adopt after graduation (Pimkhaokham et al. 2024; Mattheos et al. 2009). Rather than training in the use of new devices to perform similar tasks, the digital workflow requires an extension of education to a wider array of skills and competences. For example, as digital displays become central to both diagnostic planning and surgical execution, new essential cognitive skills are emerging at the intersection of clinical expertise and human‐computer interaction (Silvestri et al. 2024). Conducting treatment planning and execution of surgical procedures through screen‐displayed 3‐dimensional representations such as computer‐assisted design implant planning software (CAD‐IPS) or cone beam computer tomography (CBCT), can influence the development of important cognitive competences such as the spatial representation ability of young dentists (Yao et al. 2019). At the same time, emerging evidence suggests that the digital technologies utilized in CAIS have significant educational potential in the training and development of critical skills (Yao et al. 2019; Teparrukkul et al. 2024; Zhan et al. 2021; Zhong et al. 2024; Spille et al. 2022; Wang et al. 2024). Early educational interventions in undergraduate curriculum with CAD software instead of conventional training with waxing of teeth have yielded improved outcomes (Chiang et al. 2021; Mino et al. 2022), while simulation‐lab training of undergraduate (Teparrukkul et al. 2024) and postgraduate (Kunakornsawat et al. 2023) dental students with dynamic CAIS (d‐CAIS) was shown to benefit their overall development of surgical skills and competences. On the other hand, technologies such as static CAIS (s‐CAIS) have shown little educational value when utilized in the undergraduate dental curriculum (Søndergaard et al. 2021). The learning curves of different CAIS technologies appear to differ significantly (Pimkhaokham et al. 2022). Thus, the challenge dental educators worldwide face today is multifold: to design effective and clinically relevant education to support the efficient transition of practicing dentists to modern digital workflows, while also to ensure the validity of the existing dental curricula to train future dentists in the technologies and workflows they are most likely to work with after graduation.

The aim of the present white paper is to identify and discuss the educational implications introduced by the use of CAIS, and to synthesize the best available scientific evidence with best practice and expert opinion. In particular, the paper will focus on questions related to the best practices for teaching CAIS to novice and experienced dentists, related competences and learning curves, as well as the potential value of such technologies as instruments for teaching and learning. This way, the paper aspires to support dental educators in their mission to design efficient and relevant educational interventions in the dental curriculum, but also within the spectrum of CPD.

## 2 Materials and Methods

A literature review was conducted in major electronic databases (MEDLINE, EMBASE, CENTRAL, and Google Scholar) aiming to identify studies assessing education with the use of CAIS. “Education,” “teaching and learning,” “dental implant,” and “CAIS” were applied as search terms. Studies in English published up to February 28, 2025, were screened, and relevant data were extracted where available. Both clinical and preclinical studies were included and reviewed. The number of identified studies investigating education or teaching and learning for CAIS is shown in Table 1.

**Table 1:** The number of studies investigating education or teaching and learning for CAIS (database search updated 28th February 2025).

CAIS approach	Simulation studies	Clinical studies
**Non‐guided CAIS**	***n*** = ***1*** Jorba‐Garcia et al. (2019)	
**Static CAIS** **(s‐CAIS)**	***n*** = **1** Reiff et al. (2024)	***n*** = ***3*** Cassetta and Bellardini (2017) Cassetta et al. (2020) Søndergaard et al. (2021)
**Dynamic CAIS** **(d‐CAIS)**	***n*** = ***8*** Carrico et al. (2024) Kundaechanont et al. (2024) Spille et al. (2022) Sun et al. (2018) Sun et al. (2019) Golob Deeb et al. (2019) Kunakornsawat et al. (2023)	
**Robotic CAIS** **(r‐CAIS)**	***n*** = ***1*** Zhuang et al. (2025)	
**Non‐guided CAIS** **and d‐CAIS**	***n*** = ***3*** Teparrukkul et al. (2024) Zhong et al. (2024) Zhan et al. (2021)	***n*** = ***1*** Block et al. (2017)
**s‐CAIS and d‐CAIS**	***n*** = ***1*** Wang et al. (2023)	
**Non‐guided CAIS,** **s‐CAIS and d‐CAIS**	***n*** = ***1*** Werny et al. (2025)	

The findings of the literature review were discussed in a panel and further synthesized with 14 expert clinicians with extensive clinical, teaching and research experience in the application of CAIS. The results and discussions focused on four main domains relevant to education, which were then organized and presented under the four main questions presented.

**Figure 1:**
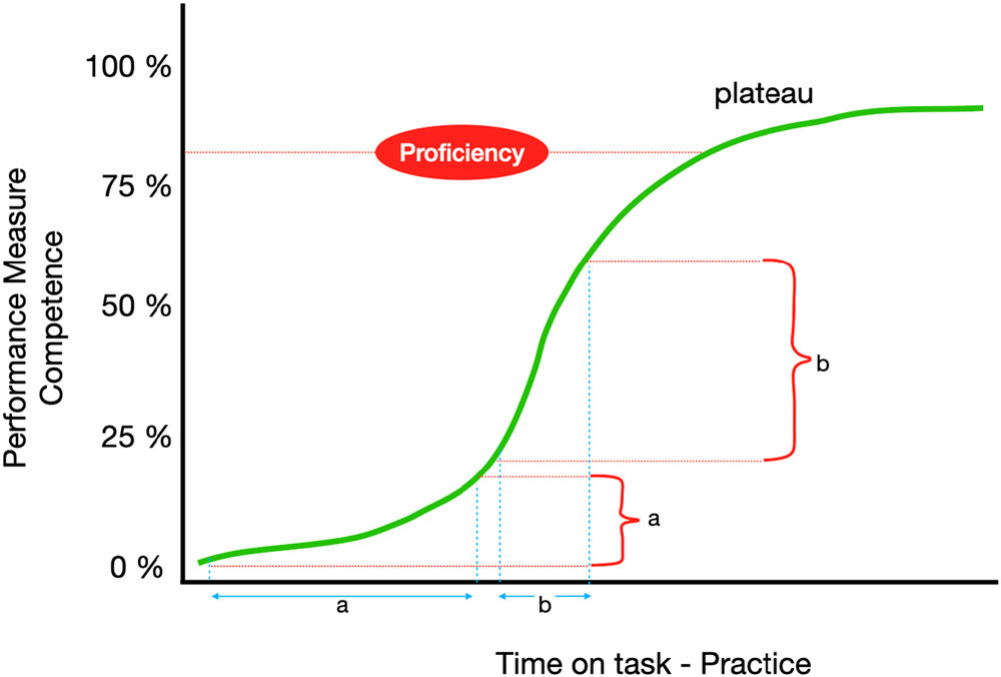
Graphic representation of a learning curve with typical elements: a) increasing returns curve, characteristic of a complex task where learning is slow and performance increases slowly with time/effort b) diminishing returns curve, characteristic of simpler tasks or stages where performance increases rapidly with time/effort. Proficiency: level of performance/competence benchmarked as proficient operator. Plateau: stage in the learning process where time on task yields only a minor increase in performance/competence.

1. **What is the value of guided CAIS in dental education?**
**Summary:**
  Guided CAIS enables more accurate implant placement and can enhance confidence among novice surgeons. d‐CAIS, in particular, might have potential for the development of wider surgical competences in implant dentistry, while the value of s‐CAIS as a wider educational tool might be limited.
**Explainer:**
  Digital 3‐dimensional technologies such as CBCT, intra‐oral and extra‐oral scanners, and CAD‐IPS, provide on‐screen interactive visualization of the anatomical structures, offering significant teaching and learning advantages over conventional 2D radiographic images and physical models. CAD‐IPS is widely used in dental implant education (Kihara et al. 2017; Nkenke et al. 2012) as it helps students and practicing dentists to better understand anatomical structures related to the intended intervention, improve spatial representation skills, while also supporting comprehensive and interdisciplinary treatment planning. Moreover, a potential “novelty effect” of guided CAIS may enhance student engagement (Elston 2021), facilitating the comprehension of clinical workflows and acquisition of skills, which could also extrapolate to non‐guided surgery (Teparrukkul et al. 2024; Zhan et al. 2021; Zhong et al. 2024; Spille et al. 2022). Whether the teaching of guided CAIS can help students develop wider surgical competences is an emerging hypothesis. One pilot study assessing training with various guided techniques (s‐CAIS, d‐CAIS, and traditional acrylic‐based surgical stent), has shown only marginal improvements in accuracy with non‐guided CAIS following the training (Carrico et al. 2024). However, simulation studies on novice students trained specifically with d‐CAIS have reported substantial improvements in students' confidence and skills, which also extended to their performance with non‐guided implant placement (Teparrukkul et al. 2024; Zhan et al. 2021; Zhong et al. 2024; Spille et al. 2022). At the same time, undergraduate dental students who used s‐CAIS for implant placement in patients found it to be an inferior learning experience to the freehand placement, although higher accuracy was reached (Søndergaard et al. 2021).
  Guided CAIS can also transform the training and execution of complex surgical interventions such as those involving sinus augmentation. Such procedures, particularly with transcrestal approach, often called “blind” surgeries, could be transformed by the use of d‐CAIS, in both clinical and preclinical settings. The use of a navigation system will allow a novice operator to visualize and precisely control the trajectory of all instruments while facilitating effective supervision of the procedure (Wang et al. 2024).
**Critique:**
  The use of CAIS as an educational tool for novice surgeons has been assessed in a few studies with typically small sample sizes and mainly focused on accuracy as an outcome measure of implant placement (Teparrukkul et al. 2024; Zhan et al. 2021; Zhong et al. 2024; Spille et al. 2022). It is reasonable to assume that technologies that include 3‐dimensional visualization either in the stage of planning with CAD‐IPS or in the stage of execution with d‐CAIS, would enhance several competencies related to implant surgery. This is, however, more likely to be achieved as part of wider educational strategies, rather than small‐scale isolated instances of training. This might require curricula with a long‐term and well‐planned competency development strategy in a comprehensive digital workflow, to which training with CAIS could significantly contribute.
2. **Should beginners be trained in guided CAIS?**
**Summary:**
  The sequence of learning—starting with guided or non‐guided CAIS—might be less critical than ensuring implant surgeons are proficient in both guided and non‐guided CAIS, and competent to select the most suitable surgical approach based on the indications and the preferences of the patient.
**Explainer:**
  It has been suggested that guided CAIS should be reserved for experienced practitioners and not beginners, as guided CAIS will enhance performance rather than compensate for a lack of fundamental skills (Cassetta et al. 2020). At the same time, different guided CAIS approaches might present different learning potentials. Static CAIS, enforcing a prescribed pathway for the osteotomy and implant placement, as well as obscuring vision, will not contribute to the development of critical surgical skills of spatial representation and alignment of the osteotomy and implant position. On the other hand, d‐CAIS being a navigated freehand implant placement technique allows the surgeon to practise the full array of surgical competences, while also adding a cognitive layer of skills by providing spontaneous spatial feedback. This on‐display real‐time information, however, might add cognitive (Yao et al. 2019) or ergonomic (James et al. 2011) complexity that could be challenging for the novice surgeon or trainee.
  In the absence of solid evidence, the authors would advocate a long‐term, teaching approach combining different approaches to support the development of surgical competencies in implant dentistry with reference to Fitts and Posner's Three‐Stage Model on how individuals acquire motor skills (Fitts and Posner 1967). For novice surgeons, preclinical simulation training can start with conventional implant placement to establish an understanding of the procedures and devices. Training with s‐CAIS at this preclinical level might contribute little to the development of surgical competences, other than familiarity with the procedure and desired outcomes. On the other hand, training with d‐CAIS could enhance the associative phase, whereby the operator refines operative skills and becomes more efficient and accurate in execution through practice and feedback. Lastly, at the autonomous stage, s‐CAIS or d‐CAIS can be utilized to increase efficiency, minimize human errors, and ensure consistency in outcomes such as immediate restoration or loading in the esthetic zone.
  Guided CAIS cannot substitute supervision for the novice surgeon or compensate for the lack of training and experience. The lack of supervisors or experienced surgeons with a good understanding of guided CAIS, could be a common barrier for the training of novice surgeons in the use of such technologies.
**Critique:**
  There is little evidence to support decisions with regard to the appropriate sequence of CAIS approaches in training of novice implant surgeons. At the same time, the end point of every curriculum in implant surgery should be a surgeon who is proficient in both guided and non‐guided CAIS for the respective level of complexity. The inherent educational value of the different CAIS approaches might dictate their role in competency development. Early exposure to d‐CAIS could enhance the learning process of novice surgeons, while training in s‐CAIS might be more suitable for improving efficiency of experienced surgeons in straightforward or complex workflows.
3. **Is the “learning curve” different for static, dynamic, and robotic‐CAIS? What are the educational implications and best teaching strategies?**
  S**ummary:**
  Mastering d‐CAIS involves developing a wide array of cognitive and clinical skills, demanding a gradual path to competency acquisition and continuous training. S‐CAIS, on the other hand, appears faster to master and involves a narrower skillset, primarily related to the procedure itself. The essential skills and competencies for a dentist to successfully engage with robotic CAIS (r‐CAIS) have not been clearly articulated yet.
**Explainer:**
  The term “learning curve” refers to the gradual development of skills and competencies as a result of training and practice on a specific task. Proposed by Wright in 1936 (Wright 1936), the learning curve usually takes the form of a graphic representation and illustrates the correlation between time on task and the level of competence achieved from novice to mastery of the respective skill (Figure 1) (Hernandez et al. 2012). Mastery of complex procedures such as CAIS might require learning of multiple skills and competencies, but with the use of clear learning objectives and end points, learning curves can offer a strategic overview of the entire learning process, identify the “pain points” the learners experience while in training and design more effective educational interventions. Few studies are available investigating the learning process of CAIS, while the outcome measures commonly utilized to assess competencies are the accuracy of implant placement and the overall duration of the procedure.
  Simulation studies (Teparrukkul et al. 2024; Jorba‐García et al. 2019) have demonstrated a significant difference in accuracy between experienced and novice implant surgeons when using d‐CAIS (Jorba‐García et al. 2019), which highlights the value of experience with conventional implant surgery before engaging with d‐CAIS. On the other hand, d‐CAIS is a novel surgical approach which involves understanding navigation technology and simultaneously maintaining focus on real‐time imaging, the operating field, and the patient. Thus, even surgeons experienced with conventional implant surgery will encounter a learning curve with d‐CAIS (Block et al. 2017). This learning curve might be very different in simulation training – where experienced surgeons appear to reach the “plateau” in accuracy after as little as 5–6 cases (Teparrukkul et al. 2024; Golob Deeb et al. 2019; Sun et al. 2019; Wang et al. 2023; Werny et al. 2025) – than in actual clinical practice. In contrast, the learning plateau for accuracy might be significantly higher in actual clinical settings, with one study reporting the surgeon's accuracy with d‐CAIS to continue improving over the first 20 surgeries (Block et al. 2017), suggestive of a “increasing returns” learning curve (Figure 1) (Hernandez et al. 2012). Accuracy is just one of the indicators to define surgical competence with this technology. Some reports have also emphasized that ergonomics, the position of the implant site, and the dominant hand of the surgeon might have implications for the learning curve and accuracy achieved (Kundaechanont et al. 2024), with posterior implants in the opposing side of the dominant hand being the most difficult for the operator (Golob Deeb et al. 2019). In contrast, the precision of s‐CAIS is not significantly influenced by the level of operators' experience in simulation studies. Novice users could achieve results comparable to those of experienced clinicians in terms of accuracy (Reiff et al. 2024), while the learning curve for s‐CAIS appears more of the “diminishing returns type” (Cassetta et al. 2020; Wang et al. 2023; Cassetta and Bellardini 2017).
  As seen with precision, learning curves of d‐ and s‐CAIS show similar characteristics when duration of the surgery was assessed as the main outcome (Golob Deeb et al. 2019; Sun et al. 2019; Wang et al. 2023; Sun et al. 2018). In one pilot study with d‐CAIS, males showed marginally shorter duration of implant placement and better 3D angulation than females (Golob Deeb et al. 2019), something that the authors associated with higher experience with computer gaming in this group. As for the teaching strategies, a distributed d‐CAIS training program (3 training sessions over 3 days) for novice students resulted in a shorter operation duration with a similar level of accuracy compared to a massed training program (3 training sessions over the same day) (Kunakornsawat et al. 2023).
  Developing competences with r‐CAIS remains largely unexplored. A pilot simulation trial with a semi‐active, task‐autonomous CAIS‐robot showed that operators with no previous experience with r‐CAIS could achieve high accuracy in 8 consecutive implant placements (Zhuang et al. 2025). Still, the essential competences required to safely supervise a CAIS robot were not articulated.
**Critique:**
  Current research has documented significant differences between s‐ and d‐CAIS with regard to the teaching and learning process. The teaching of s‐CAIS appears closer to teaching of a procedure related to a narrow array of competencies, while the teaching of d‐CAIS targets a wider set of cognitive and clinical skills, spreading over a longer period of time. At the same time, practice with d‐CAIS might have a higher educational value for novice surgeons. Thus, it can be recommended to proceed with the teaching of s‐CAIS to surgeons already competent with freehand surgery, while d‐CAIS might have educational value at earlier stages of surgical skills development. However, the strategies that would work best when designing such an education remain anecdotal. The few available studies on learning curves are mainly conducted on brief simulation training and focus on simple outcomes as surrogates of competence, such as accuracy of implant placement and duration of the procedure. Future studies might be best directed to investigate how competency grows in actual clinical settings and also prospectively assess outcomes related to the surgeon's learning experience, user‐friendliness, and efficacy of the device and setup, as well as identify barriers and “pain points” for the learner. Education and training with r‐CAIS remain largely unexplored. The essential competences and knowledge for a dentist to supervise the operation of a semi‐autonomous CAIS robot are not articulated and should not be understated. Although many would consider that supervising a robotic arm conducting an osteotomy by means of a foot pedal is relatively simple, the fact remains that such robotic operations require a highly complex setup, involve very sensitive and complex technologies, and as of today are typically performed in the presence of a robotic engineer. Even for the experienced clinicians, operating a CAIS robot requires a deep understanding of the process and troubleshooting skills to early identify and manage potential complications, many of which are currently not described or unknown. Thus, the actual skillset essential for a dentist to supervise such procedures is yet to be defined, and the authors strongly believe that the role of the dentist in r‐CAIS is far more complex than depicted in the limited clinical trials available in this field. Future growth in the field of r‐CAIS would necessitate a clear description of the role and competencies of the dentist, as well as foster the frameworks of essential training in the discipline.
4. **Which are currently the major challenges for designing and implementing effective education and training with guided CAIS?**
**Summary:**
  Incomplete transition to digital workflows in implant dentistry is usually associated with a lack of understanding of CAIS technologies and constitutes a common barrier for their implementation. At the institutional level, the lack of experienced faculty members, the appropriate timing of such education and its implementation in existing curricula are also challenges frequently encountered in university environments. Finally, other barriers included high cost and availability of digital devices, vendor‐specific closed systems with limited interoperability, and ethical or legal concerns regarding the increasing autonomy of surgical technologies.
**Explainer:**
  A wide array of factors can influence dentists, clinics, and universities to adopt digital technologies, such as increased efficiency over traditional methods, perceived influence on treatment quality, personal and professional orientation, and influence from peers and external groups (van der Zande et al. 2013). At the same time, clinicians experienced with conventional non‐guided implant placement may often view guided CAIS as unnecessary or even a threat to the development of manual expertise. They may see no benefits in spending time to learn a new technology as suggested in “The Technology Acceptance Model” (Figure 2) (Marikyan and Papagiannidis 2024). On the other hand, availability of digital devices and often an initial high investment cost can make it difficult for institutions or clinics with limited budget to overhaul their existing educational frameworks and integrate CAIS into curricula. The cost of acquiring and maintaining human expertise, devices, and software for full digital workflow is significant. These investments may be questioned, especially when conventional practice is perceived as adequate.
  **Figure 2:**
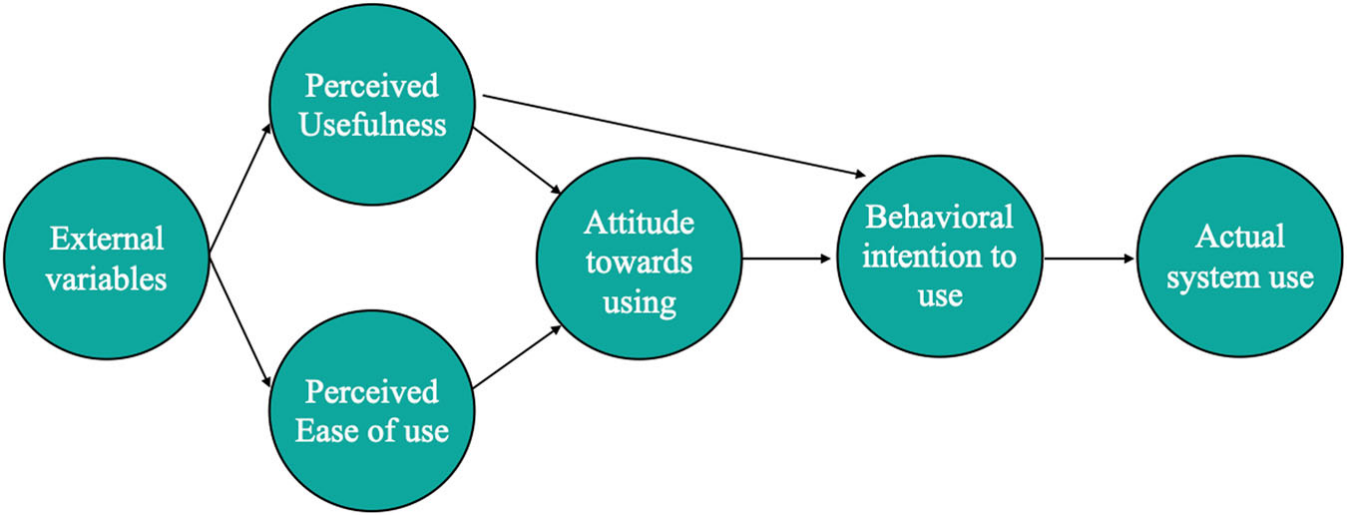
The Technology Acceptance Model suggests that the adoption of a new technology is influenced by users' behavioral intentions, which are, in turn, shaped by their perceptions of the technology's usefulness for completing tasks and its ease of use.
  The fragmentation of the CAIS development, as well as the overall digital dentistry market is another frequently encountered barrier. Guided CAIS is rapidly evolving, with different manufacturers developing proprietary software, hardware, and workflows. This diversity frequently creates inconsistencies in the way students and professionals are trained (Zhu et al. 2024). The reliance on vendor‐specific resources can lead to a fragmented clinical workflow, complicate the learning experience, and potentially bias education toward particular products or techniques where partnership or sponsorship is available.
  Another significant concern about guided CAIS could be the regulatory, ethical, and legal considerations, which are particularly relevant to the increasing levels of autonomy introduced by surgical robots. Surgical robots have been categorized into six levels based on autonomy of operation: 0‐no autonomy, 1‐robot assistance, 2‐task autonomy, 3‐conditional autonomy, 4‐high autonomy, 5‐full autonomy (Yang et al. 2017). Each level of autonomy will introduce different clinical and ethical challenges; there is however at present no clear understanding of the essential training required for a clinician to successfully operate or supervise CAIS robots at each of them.
**Critique:**
  Although the emerging digital workflow can offer major advantages for the overall treatment outcomes and also significantly benefit the patient, the transition from the conventional analog practice is at its early stages. Much of the skepticism towards CAIS originates from the perception that it is just another method of implant placement and the increased accuracy might be of limited benefit to an experienced clinician. The key for the majority to embrace a new technology is when unprecedented capabilities are proven, delivering fundamental value that resonates even with nontechnical stakeholders. Rather than another approach of implant placement, guided CAIS should be seen as an essential link in an advanced workflow: a digital workflow delivering higher efficiency and minimally invasive treatment. If this core value is not obvious to the majority, then a “chasm” exists between the early adopters and the majority, limiting the use of the technology to the inventors and early adopters. Documenting with research and publication the tangible benefits of guided CAIS could help alleviate skepticism and reduce the chasm of the technology adoption lifecycle (Figure 3) (Moore and McKenna 1999).
  **Figure 3:**
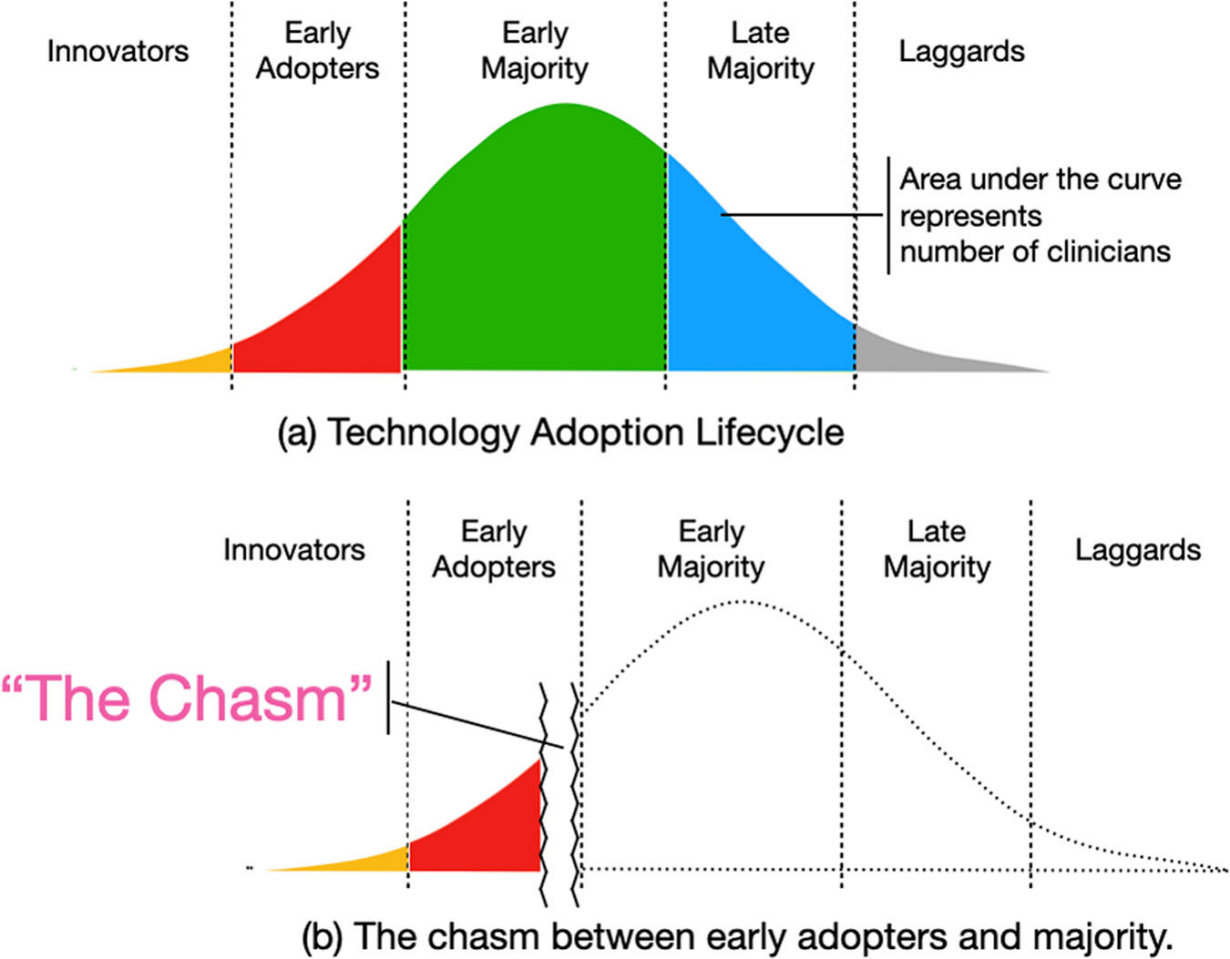
(a) A theoretical curve of the adoption of technology lifecycle representing how the target population adopts innovations over time, segmented by psychological readiness. Colored areas represent the proportion of people using the new technology. At the beginning, only a small group of innovators (yellow area) have access and utilize the new technology, then more early adopters (red area) see the potential and adopt it, followed by an exponential growth when the early majority (green area) and late majority (blue area) adopt the technology. Lastly, the laggards (gray area) are the last ones to join an established technology. (b) The chasm that is often created between early adopters and the early majority, when a clear core value is not yet obvious to the majority.
  At the same time, the lack of interoperability of CAIS with many established workflows is a major drawback for the clinician. The inability to freely choose the Computer‐aided Design Implant Planning Software (CAD‐IPS) and combine it with the implant system and the d‐CAIS system of choice is a major obstacle repeatedly cited by most clinicians, who often have to resort to “digital acrobatics” and hacks to continue using their habitual software, devices, and implants. If industry and manufacturers would like to best serve the clinicians, the major emerging request is to focus on interoperability of their systems through common standards, rather than continuously trying to lock their users in proprietary devices, formats, and workflows.
  Finally, regulatory frameworks typically lag behind technological progress, resulting in practical challenges concerning ethics and liability. Devices essential for CAIS are being approved by regulators at different levels, implying different degrees of responsibility for the operator. Thus, although the role and liability of the clinician is clearer when operating nonautonomous devices such as d‐CAIS systems and level 0 CAIS robots, this becomes increasingly vague as the autonomy level of the device increases, as currently most commercially available CAIS robots are at autonomy Levels 1 and 2. The further “augmentation” of related software and procedures with machine learning and AI‐assisted components, might further blur the margins of responsibility between manufacturers and clinicians, thus also necessitating the update of the competences and training in these aspects.

## 3 Discussion

The integration of CAIS into dental education appears to interweave with the challenges and opportunities of transitioning to a digital workflow in clinical practice. As a result, it becomes apparent that CAIS cannot be taught in isolation of the wider digital practice. To fully harness the potential of guided CAIS in education, it is envisioned that a structured, long‐term curricula encompassing comprehensive digital workflows will be required with multi‐stakeholders input.

Digital 3D imaging, such as CBCT and intra‐oral scanners, can provide significant educational benefits by enhancing visualization of anatomical structures and improving spatial representation skills. Furthermore, the use of CAD‐IPS can help link theory and practice, allowing the learner to understand and apply the evidence‐based design principles of implant therapy. The use of guided CAIS, thereafter, not only enables more accurate implant placement but when used strategically could also enhance the confidence of students and improve their overall surgical skills. In particular, d‐CAIS appears to support the development of a broader range of cognitive and technical skills, whereas s‐CAIS may be limited to improving procedural accuracy without fostering deeper surgical competency.

There is no conclusive evidence to suggest a definitive sequence between guided and non‐guided CAIS approaches. What, however, emerges as an essential end point is to train surgeons who master both approaches, competent to select the most appropriate method depending on indications, patients' needs and clinical circumstances. The learning curves associated with s‐CAIS and d‐CAISalso differ significantly, with important implications for education and training strategies. An educational model is therefore advocated where both guided and non‐guided approaches are combined: for novice surgeons, preclinical simulation with conventional implant placement to establish fundamental skills, followed by d‐CAIS to foster spatial awareness and cognitive competences. In clinical settings, first expose novice surgeons to straightforward non‐guided implant placement, before progressing to d‐CAIS. S‐CAIS may be incorporated later in clinical practice to improve procedural efficiency without substituting the need for fundamental surgical experience. For experienced surgeons, preclinical training is essential to developing spatial representation and hand‐eye coordination skills before applying d‐CAIS in clinical setting. Finally, r‐CAIS is a relatively new approach, without an established set of essential competences and thus outside the domain of conventional education. The complexity involved in r‐CAIS procedures is likely underestimated at present, especially when it comes to robots with task autonomy. A complete educational framework for r‐CAIS would be necessary before such technology can reach wider adoption. Such a framework should include a clear description of the essential competencies and responsibilities of the clinicians, describe the training and education pathway required to reach proficiency, and also address several ethical and legal challenges.

Despite the educational value of CAIS, several major challenges hinder its effective implementation in training programs, especially at the institutional level. At the same time, educational research in support of such implementation efforts is at an early stage. The majority of studies on teaching and learning with CAIS are focused on assessing procedures and not workflows, with common outcome measures of competency being accuracy of implant placement or duration of the procedure. Future research would need to extend from the procedure to the workflow, link simulation practice to clinical care, and include a wider array of outcomes that can help better identify the competencies that should be attained by undergraduate and postgraduate dental education. The primary objective of education remains the development of a competent and reflective practitioner, empowered by technology, who can devise and execute the optimal treatment for the benefit of individual patients.

## Author Contributions

Conceptualization: Bilal Al‐Nawas and Nikos Mattheos. Data curation: Lin Jing Uei and Xin Hui Yeo. Formal analysis: Lin Jing Uei, Xin Hui Yeo, Bilal Al‐Nawas, and Nikos Mattheos. Investigation: Xin Hui Yeo and Lin Jing Uei. Methodology: Bilal Al‐Nawas, Nikos Mattheos, Lin Jing Uei, and Xin Hui Yeo. Project administration: Bilal Al‐Nawas and Nikos Mattheos. Resources: Bilal Al‐Nawas and Nikos Mattheos. Supervision: Bilal Al‐Nawas, Nikos Mattheos. Validation Xin Hui Yeo, Lin Jing Uei, Yiu Yan Leung, and George Pelekos. Visualization: Lin Jing Uei, Xin Hui Yeo, and Nikos Mattheos. Writing – original draft: Lin Jing Uei, Xin Hui Yeo, and Nikos Mattheos. Writing – review and editing: Lin Jing Uei, Xin Hui Yeo, Nikos Mattheos, Yiu Yan Leung, George Pelekos, and Bilal Al‐Nawas.

## Conflicts of Interest

The authors declare no conflicts of interest.

## Data Availability

Data sharing is not applicable to this article as no datasets were generated or analyzed during the current study.

## References

[cre270197-bib-0001] Amorfini, L. , M. Migliorati , S. Drago , and A. Silvestrini‐Biavati . 2017. “Immediately Loaded Implants in Rehabilitation of the Maxilla: A Two‐Year Randomized Clinical Trial of Guided Surgery Versus Standard Procedure.” Clinical Implant Dentistry and Related Research 19, no. 2: 280–295. 27790821 10.1111/cid.12459

[cre270197-bib-0002] Block, M. , R. Emery , K. Lank , and J. Ryan . 2017. “Implant Placement Accuracy Using Dynamic Navigation.” The International Journal of Oral & Maxillofacial Implants 32, no. 1: 92–99. 27643585 10.11607/jomi.5004

[cre270197-bib-0003] Carrico, C. , L. Skrjanc , D. Kanduti , G. Deeb , and J. G. Deeb . 2024. “Effect of Guided Implant Placement Learning Experiences on Freehand Skills: A Pilot Study.” Clinical and Experimental Dental Research 10, no. 2: e878. 38506282 10.1002/cre2.878 PMC10952114

[cre270197-bib-0004] Cassetta, M. , F. Altieri , M. Giansanti , M. Bellardini , G. Brandetti , and L. Piccoli . 2020. “Is There a Learning Curve in Static Computer‐Assisted Implant Surgery? A Prospective Clinical Study.” International Journal of Oral and Maxillofacial Surgery 49, no. 10: 1335–1342. 32217033 10.1016/j.ijom.2020.03.007

[cre270197-bib-0005] Cassetta, M. , and M. Bellardini . 2017. “How Much Does Experience in Guided Implant Surgery Play a Role in Accuracy? A Randomized Controlled Pilot Study.” International Journal of Oral and Maxillofacial Surgery 46, no. 7: 922–930. 28366450 10.1016/j.ijom.2017.03.010

[cre270197-bib-0006] Chiang, H. , A. Staffen , A. A. Abdulmajeed , C. Janus , and S. Bencharit . 2021. “Effectiveness of CAD/CAM Technology: A Self‐Assessment Tool for Preclinical Waxing Exercise.” European Journal of Dental Education 25, no. 1: 50–55. 33448597 10.1111/eje.12576

[cre270197-bib-0007] Dragan, I. F. , M. Pirc , C. Rizea , J. Yao , A. Acharya , and N. Mattheos . 2019. “A Global Perspective on Implant Education: Cluster Analysis of the “First Dental Implant Experience” of Dentists From 84 Nationalities.” European Journal of Dental Education 23, no. 3: 251–265. 30710398 10.1111/eje.12426

[cre270197-bib-0008] Elston, D. M. 2021. “The Novelty Effect.” Journal of the American Academy of Dermatology 85, no. 3: 565–566. 34153390 10.1016/j.jaad.2021.06.846

[cre270197-bib-0009] Fitts, P. M. , and M. I. Posner . 1967. Human Performance. Brooks/Cole Publishing Company.

[cre270197-bib-0010] Golob Deeb, J. , S. Bencharit , C. K. Carrico , et al. 2019. “Exploring Training Dental Implant Placement Using Computer‐Guided Implant Navigation System for Predoctoral Students: A Pilot Study.” European Journal of Dental Education 23, no. 4: 415–423. 31141291 10.1111/eje.12447

[cre270197-bib-0011] Hernandez, J. M. , L. A. Humphries , W. B. Keeling , et al. 2012. “Robotic Lobectomy: Flattening the Learning Curve.” Journal of Robotic Surgery 6: 41–45. 27637978 10.1007/s11701-011-0275-6

[cre270197-bib-0012] James, D. R. C. , F. Orihuela‐Espina , D. R. Leff , et al. 2011. “The Ergonomics of Natural Orifice Translumenal Endoscopic Surgery (NOTES) Navigation in Terms of Performance, Stress, and Cognitive Behavior.” Surgery 149, no. 4: 525–533. 21295807 10.1016/j.surg.2010.11.019

[cre270197-bib-0013] Joda, T. , W. Derksen , J. G. Wittneben , and S. Kuehl . 2018. “Static Computer‐Aided Implant Surgery (s‐CAIS) Analysing Patient‐Reported Outcome Measures (PROMs), Economics and Surgical Complications: A Systematic Review.” Clinical Oral Implants Research 29, no. Suppl 16: 359–373. 30328203 10.1111/clr.13136

[cre270197-bib-0014] Jorba‐García, A. , R. Figueiredo , A. González‐Barnadas , O. Camps‐Font , and E. Valmaseda‐Castellón . 2019. “Accuracy and the Role of Experience in Dynamic Computer Guided Dental Implant Surgery: An In‐Vitro Study.” Medicina oral, patologia oral y cirugia bucal 24, no. 1: 76. 10.4317/medoral.22785 PMC6344002 30573712

[cre270197-bib-0015] Kihara, H. , J. Sun , M. Sakai , S. Nagai , and J. Da Silva . 2017. “A Survey of Dental Implant Instruction in Predoctoral Dental Curricula in North America.” Journal of Dental Education 81, no. 9: 1085–1090. 28864790 10.21815/JDE.017.065

[cre270197-bib-0016] Kunakornsawat, W. , P. Serichetaphongse , S. Arunjaroensuk , B. Kaboosaya , N. Mattheos , and A. Pimkhaokham . 2023. “Training of Novice Surgeons Using Dynamic Computer Assisted Dental Implant Surgery: An Exploratory Randomized Trial.” Clinical Implant Dentistry and Related Research 25, no. 3: 511–518. 36958848 10.1111/cid.13201

[cre270197-bib-0017] Kundaechanont, P. , P. Serichetaphonges , A. Pimkhaokham , and W. Chengprapakorn . 2024. “Comparison of Learning Curve for Anterior and Posterior Implant Placement Using Dynamic Computer‐Assisted Surgical Implant Placement in Novice and Experienced Operators: An In Vitro Trial.” Journal of Prosthetic Dentistry: S0022‐3913(24)00720‐0. 10.1016/j.prosdent.2024.10.025. 39617665

[cre270197-bib-0018] Marikyan, D. , and S. Papagiannidis . 2024. “Technology Acceptance Model: A review.” In TheoryHub Book, edited by S. Papagiannidis . Newcastle University.

[cre270197-bib-0019] Mattheos, N. , T. Albrektsson , D. Buser , et al. 2009. “Teaching and Assessment of Implant Dentistry in Undergraduate and Postgraduate Education: A European Consensus.” European Journal of Dental Education 13, no. Suppl 1: 10–17. 19281510 10.1111/j.1600-0579.2008.00556.x

[cre270197-bib-0020] Mattheos, N. , I. Vergoullis , M. Janda , and A. Miseli . 2021. “The Implant Supracrestal Complex and Its Significance for Long‐Term Successful Clinical Outcomes.” International Journal of Prosthodontics 34, no. 1: 88–100. 33570524 10.11607/ijp.7201

[cre270197-bib-0021] Mino, T. , Y. Kurosaki , K. Tokumoto , et al. 2022. “Rating Criteria to Evaluate Student Performance in Digital Wax‐Up Training Using Multi‐Purpose Software.” Journal of Advanced Prosthodontics 14, no. 4: 203–211. 36105880 10.4047/jap.2022.14.4.203 PMC9444485

[cre270197-bib-0022] Moore, G. A. , and R. McKenna . 1999. Crossing the Chasm: Marketing and Selling High‐tech Products to Mainstream Customers. HarperBusiness.

[cre270197-bib-0023] Morton, D. , D. Wismeijer , S. Chen , et al. 2023. “Group 5 ITI Consensus Report: Implant Placement and Loading Protocols.” Clinical Oral Implants Research 34, no. Suppl 26: 349–356. 37750529 10.1111/clr.14137

[cre270197-bib-0024] Nkenke, E. , E. Vairaktaris , A. Bauersachs , et al. 2012. “Acceptance of Virtual Dental Implant Planning Software in an Undergraduate Curriculum: A Pilot Study.” BMC Medical Education 12, no. 1: 90. 23020863 10.1186/1472-6920-12-90 PMC3511246

[cre270197-bib-0025] Pedrinaci, I. , A. Hamilton , A. Lanis , M. Sanz , and G. O. Gallucci . 2024. “The Bio‐Restorative Concept for Implant‐Supported Restorations.” Journal of Esthetic and Restorative Dentistry 36: 1516–1527. 39210698 10.1111/jerd.13306

[cre270197-bib-0026] Pimkhaokham, A. , J. Chow , A. Pozzi , S. Arunjaroensuk , K. Subbalehka , and N. Mattheos . 2024. “Computer‐Assisted and Robotic Implant Surgery: Assessing the Outcome Measures of Accuracy and Educational Implications.” Clinical Oral Implants Research 35, no. 8: 939–953. 37994685 10.1111/clr.14213

[cre270197-bib-0027] Pimkhaokham, A. , S. Jiaranuchart , B. Kaboosaya , S. Arunjaroensuk , K. Subbalekha , and N. Mattheos . 2022. “Can Computer‐Assisted Implant Surgery Improve Clinical Outcomes and Reduce the Frequency and Intensity of Complications in Implant Dentistry? A Critical Review.” Periodontology 2000 90, no. 1: 197–223. 35924457 10.1111/prd.12458 PMC9805105

[cre270197-bib-0028] Reiff, F. S. , C. Bischoff , H. Woelfler , and S. Roehling . 2024. “Influence of Clinical Expertise and Practical Experience on Transfer Accuracy in Guided Dental Implant Placement – An In Vitro Study.” Oral and Maxillofacial Surgery 28, no. 4: 1491–1500. 38914846 10.1007/s10006-024-01269-4 PMC11480191

[cre270197-bib-0029] Silvestri, F. , N. Odisho , A. Kumar , and A. Grigoriadis . 2024. “Examining Gaze Behavior in Undergraduate Students and Educators During the Evaluation of Tooth Preparation: An Eye‐Tracking Study.” BMC Medical Education 24, no. 1: 1030. 39300488 10.1186/s12909-024-06019-4 PMC11414055

[cre270197-bib-0030] Spille, J. , E. Helmstetter , P. Kübel , et al. 2022. “Learning Curve and Comparison of Dynamic Implant Placement Accuracy Using a Navigation System in Young Professionals.” Dentistry Journal 10, no. 10: 187. 36285997 10.3390/dj10100187 PMC9600962

[cre270197-bib-0031] Sun, T. M. , T. H. Lan , C. Y. Pan , and H. E. Lee . 2018. “Dental Implant Navigation System Guide the Surgery Future.” Kaohsiung Journal of Medical Sciences 34, no. 1: 56–64. 29310817 10.1016/j.kjms.2017.08.011 PMC11915682

[cre270197-bib-0032] Sun, T.‐M. , H.‐E. Lee , and T.‐H. Lan . 2019. “The Influence of Dental Experience on a Dental Implant Navigation System.” BMC Oral Health 19, no. 1: 222. 31623636 10.1186/s12903-019-0914-2 PMC6798373

[cre270197-bib-0033] Søndergaard, K. , M. Hosseini , S. Storgård Jensen , R. Spin‐Neto , and K. Gotfredsen . 2021. “Fully Versus Conventionally Guided Implant Placement by Dental Students: A Randomized Controlled Trial.” Clinical Oral Implants Research 32, no. 9: 1072–1084. 34166539 10.1111/clr.13802

[cre270197-bib-0034] Teparrukkul, H. , P. Serichetaphongse , W. Chengprapakorn , S. Arunjaroensuk , N. Mattheos , and A. Pimkhaokham . 2024. “Training Outcomes of Novice Clinicians in the Use of Dynamic Computer Assisted Implant Surgery: A Prospective Comparative Study.” Journal of Dental Sciences 19: S122–S127. 39807253 10.1016/j.jds.2024.07.018 PMC11725117

[cre270197-bib-0035] Wang, J. , B. Wang , Y. Y. Liu , et al. 2024. “Recent Advances in Digital Technology in Implant Dentistry.” Journal of Dental Research 103, no. 8: 787–799. 38822563 10.1177/00220345241253794

[cre270197-bib-0036] Wang, W. , M. Zhuang , S. Li , et al. 2023. “Exploring Training Dental Implant Placement Using Static or Dynamic Devices Among Dental Students.” European Journal of Dental Education 27, no. 3: 438–448. 35579548 10.1111/eje.12825

[cre270197-bib-0037] Werny, J. G. , S. Fan , L. Diaz , et al. 2025. “Evaluation of the Accuracy, Surgical Time, and Learning Curve of Freehand, Static, and Dynamic Computer‐Assisted Implant Surgery in an In Vitro Study.” Clinical Oral Implants Research 36, no. 5: 555–565. 10.1111/clr.14403. 39835464 PMC12066894

[cre270197-bib-0038] Wright, T. P. 1936. “Factors Affecting the Cost of Airplanes.” Journal of the Aeronautical Sciences 3, no. 4: 122–128.

[cre270197-bib-0039] Yang, G. Z. , J. Cambias , K. Cleary , et al. 2017. “Medical Robotics‐Regulatory, Ethical, and Legal Considerations for Increasing Levels of Autonomy.” Science Robotics 2, no. 4: eaam8638. 10.1126/scirobotics.aam8638. 33157870

[cre270197-bib-0040] Yao, C. J. , J. Chow , W. W. S. Choi , and N. Mattheos . 2019. “Measuring the Impact of Simulation Practice on the Spatial Representation Ability of Dentists by Means of Impacted Mandibular Third Molar (IMTM) Surgery on 3D Printed Models.” European Journal of Dental Education 23, no. 3: 332–343. 30825405 10.1111/eje.12434

[cre270197-bib-0041] van der Zande, M. M. , R. C. Gorter , and D. Wismeijer . 2013. “Dental Practitioners and a Digital Future: An Initial Exploration of Barriers and Incentives to Adopting Digital Technologies.” British Dental Journal 215, no. 11: E21. 24309814 10.1038/sj.bdj.2013.1146

[cre270197-bib-0042] Zhan, Y. , M. Wang , X. Cheng , Y. Li , X. Shi , and F. Liu . 2021. “Evaluation of a Dynamic Navigation System for Training Students in Dental Implant Placement.” Journal of Dental Education 85, no. 2: 120–127. 32914421 10.1002/jdd.12399

[cre270197-bib-0043] Zhong, X. , Y. Xing , J. Yan , J. Chen , Z. Chen , and Q. Liu . 2024. “Surgical Performance of Dental Students Using Computer‐Assisted Dynamic Navigation and Freehand Approaches.” European Journal of Dental Education 28, no. 2: 504–510. 37941129 10.1111/eje.12975

[cre270197-bib-0044] Zhu, J. , W. Sun , L. Li , et al. 2024. “Accuracy and Patient‐Centered Results of Marker‐Based and Marker‐Free Registrations for Dynamic Computer‐Assisted Implant Surgery: A Randomized Controlled Trial.” Clinical Oral Implants Research 35, no. 1: 101–113. 37955359 10.1111/clr.14201

[cre270197-bib-0045] Zhuang, M. , J. Chen , B. Tao , M. Gul , F. Wang , and Y. Wu . 2025. “Exploring the Learning Curve of Dental Implant Placement Using a Task‐Autonomous Robotic System Among Young Dentists From Different Specialties – A Pilot Module Study.” Clinical Implant Dentistry and Related Research 27, no. 1: e13402. 39407090 10.1111/cid.13402

